# Functional experiences of a LINE-based chatbot and their associations with system use experience among older adults: a cross-sectional study

**DOI:** 10.3389/fpubh.2026.1792791

**Published:** 2026-04-13

**Authors:** Kuo-Mou Chung, Liang-Hsi Kung, Yu-Hua Yan

**Affiliations:** Tainan Municipal Hospital (Managed by Show Chwan Medical Care Corporation), Tainan, Taiwan

**Keywords:** chatbots, community programs, digital engagement, line, older adults, system use experience

## Abstract

**Background:**

Population aging has heightened concerns regarding social participation and active aging among older adults. Chatbot-based interventions delivered through familiar messaging platforms have increasingly been adopted to support engagement with community activity programs; however, prior research has often treated chatbots as homogeneous interventions, with limited attention to how specific functional components shape user experiences among older adults.

**Objective:**

This study aimed to examine older adults’ functional experiences with a LINE-based chatbot designed to support engagement with community activity programs and to investigate how different chatbot functions are associated with overall system use experience.

**Methods:**

A cross-sectional survey was conducted among 299 older adults who used a LINE-based chatbot implemented in community-based programs. Functional experiences were assessed across three chatbot modules: information inquiry, photo-based check-in interaction, and task achievement. Composite mean scores were calculated for each functional module and for the overall system use experience scale. Descriptive statistics, Pearson correlation analyses, and multiple linear regression analyses were performed using IBM SPSS Statistics.

**Results:**

Participants reported moderate to moderately high levels of functional experience across all chatbot modules. Pearson correlation analyses showed significant positive associations among all functional experience variables. Multiple linear regression analysis indicated that the overall model explained a large proportion of variance in overall system use experience (*R*^2^ = 0.760, adjusted *R*^2^ = 0.757, *F*(3,295) = 310.72, *p* < 0.001). Task achievement experience was the strongest predictor of overall system use experience (*β* = 0.595, *p* < 0.001), followed by information inquiry experience (*β* = 0.252, *p* < 0.001), whereas photo-based check-in experience was not significantly associated with overall system use experience (*β* = 0.066, *p* = 0.139).

**Conclusion:**

The findings suggest that different chatbot functions contribute unequally to older adults’ system use experience. Task-oriented and goal-focused functions may play a central role in shaping engagement with chatbot systems, whereas interactive features such as photo-based check-ins may serve a supplementary role. These results underscore the importance of function-specific design when developing chatbot-based interventions intended to support engagement with digital systems designed for community activity programs among older adults.

## 1 Introduction

Population aging has emerged as a major global demographic trend, accompanied by growing concerns regarding social participation, active aging, and the maintenance of wellbeing among older adults. Social participation, broadly defined as engagement in social, community, and meaningful activities, is widely recognized as a key determinant of healthy aging, contributing to psychological wellbeing, cognitive functioning, and quality of life. Prior research has consistently shown that reduced social participation among older adults is associated with loneliness, social isolation, and adverse health outcomes, highlighting the need for scalable and accessible interventions to support engagement in later life (1–3).

In recent years, conversational agents and chatbot-based interventions have gained increasing attention as digital tools for health promotion, behavior change, and psychosocial support. Multiple systematic reviews and meta-analyses have demonstrated that chatbots can effectively support lifestyle modification, chronic disease self-management, and mental health outcomes by providing timely information, personalized feedback, and interactive guidance (4–7). Evidence from mental health–focused reviews further suggests that conversational agents may alleviate psychological distress and improve wellbeing through sustained engagement and perceived support (8, 9).

Among older adults, chatbot delivery through familiar communication platforms has been identified as a critical factor influencing adoption and sustained use. Studies on voice assistants and smart speakers indicate that older adults are more willing to engage with AI-based systems that align with their everyday communication practices and minimize cognitive and technical burdens (10, 11). Reviews focusing on older populations have further shown that conversational agents may reduce loneliness and enhance perceived social support, although reported effects vary depending on interaction modality and functional design (1, 2, 12).

Despite the growing evidence base, existing studies have often conceptualized chatbots as relatively homogeneous interventions. Most evaluations focus on overall usability, acceptability, or effectiveness, without systematically disentangling the specific functional components that shape user experiences (8, 13). Recent reporting guidelines and methodological reviews have emphasized that conversational agents typically integrate multiple functional elements—such as information provision, interactive engagement, reminders, and task-oriented features—but empirical evidence remains limited regarding how these components differentially influence user experience and engagement outcomes (14).

Emerging research suggests that function-specific design plays a crucial role in determining the effectiveness of chatbot-based interventions. Task-oriented and goal-focused conversational agents have been shown to promote sustained engagement and behavioral adherence by providing structured guidance and clear performance feedback, as demonstrated in randomized controlled trials and design-focused studies (15, 16). In contrast, socially oriented or interactive features—such as conversational exchanges or media-based interactions—may enhance enjoyment or emotional connection but do not consistently translate into improved overall system use, particularly among older adults who may experience higher cognitive load or usability challenges (17, 18).

Qualitative and co-design studies further indicate that older adults’ preferences for chatbot functions are heterogeneous and context-dependent. Research involving participatory design and mixed-method approaches has shown that older users value clarity, purpose, and perceived usefulness over novelty, and that poorly aligned interaction modes may hinder engagement rather than enhance it (19–21). These findings underscore the importance of examining chatbot use at the functional level rather than treating system experience as a unitary construct.

Another limitation of prior research lies in the insufficient integration of chatbot functionality with broader concepts of social participation. While many studies examine chatbots in relation to health behavior change or mental health support, fewer explicitly situate chatbot use within community engagement or social participation frameworks relevant to older adults (3, 22). Understanding how functional experiences with chatbot systems relate to users’ overall system use experience is therefore essential for designing digital interventions that meaningfully support active aging.

To address these gaps, the present study focuses on a LINE-based chatbot developed to support community engagement among older adults. LINE is a widely used messaging platform in Taiwan and other East Asian contexts, making it a familiar and accessible medium for older users. Adopting a function-oriented perspective, this study examines older adults’ experiences across three distinct chatbot modules: information inquiry, photo-based check-in interaction, and task achievement.

Accordingly, the objectives of this study were twofold: (1) to assess older adults’ functional experiences with different modules of a LINE-based chatbot designed for community engagement, and (2) to examine the associations between these functional experiences and overall system use experience. By identifying which chatbot functions most strongly shape user experience, this study aims to inform the development of chatbot-based interventions designed to support engagement with chatbot systems used in community activity programs for older adults. By empirically assessing how these functional experiences are associated with overall system use experience, this study also responds to recent calls for more granular evaluations of conversational agent design and reporting (14).

## 2 Methods

### 2.1 Study design and participants

This study employed a cross-sectional survey design to examine older adults’ functional experiences with a LINE-based chatbot developed to support community engagement. Participants were recruited from community-based activity programs in southern Taiwan where the chatbot had been implemented as part of routine engagement and participation support. Eligibility criteria included: (1) age 60 years or older, (2) prior experience using the LINE-based chatbot for community-related activities, and (3) ability to complete a self-administered questionnaire independently or with minimal assistance. Individuals with severe cognitive impairment or who were unable to communicate effectively were excluded.

The questionnaire consisted of four constructs: Information Inquiry Experience (10 items), Photo Check-in Mechanism Experience (10 items), Task Achievement Mechanism Experience (10 items), and Overall System Use Experience (11 items). The complete questionnaire items are presented in Appendix A.

The reporting of this study followed the Checklist for Reporting Results of Internet E-Surveys (CHERRIES) guideline to enhance transparency and completeness in survey reporting (23). A total of 320 questionnaires were distributed, of which 299 valid responses were returned, yielding a response rate of 93.4%. This sample size exceeded the minimum requirements for multiple linear regression analysis and was sufficient to detect moderate effect sizes with adequate statistical power.

### 2.2 Description of the LINE-based chatbot

The chatbot examined in this study was embedded within the LINE messaging platform, a widely used communication application among older adults in Taiwan. The chatbot was designed to support community engagement by facilitating access to activity-related information, enabling participation-related interactions, and supporting task completion. Consistent with prior classification of conversational agent functionalities in digital health, the chatbot incorporated multiple functional modules rather than operating as a single uniform intervention (13, 14). Specifically, three core functional modules were examined:

#### 2.2.1 Information inquiry module

This module allowed users to retrieve information related to community activities, schedules, locations, and announcements through text-based queries. The function emphasized clarity, responsiveness, and ease of access to reduce information barriers commonly experienced by older adults.

#### 2.2.2 Photo-based check-in interaction module

This module enabled users to upload photos as a form of participation confirmation or interaction during community activities. The design aimed to promote engagement and a sense of involvement through visual interaction, while requiring basic smartphone camera and upload functions.

#### 2.2.3 Task achievement module

This module supported goal-oriented actions such as completing assigned tasks, confirming attendance, or achieving predefined participation milestones. Task completion feedback was provided through the chatbot, reinforcing goal attainment and progress tracking.

**Figure 1:**
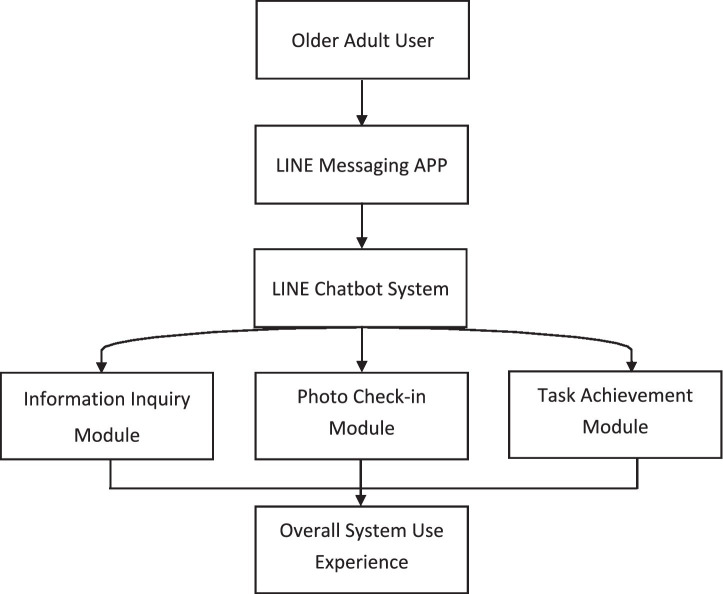
Conceptual structure of the LINE-based chatbot system used in this study. Community-dwelling older adults interacted with the chatbot through the LINE messaging platform. The system provided three functional modules: information inquiry, photo-based check-in interaction, and task achievement.

**Figure 2:**
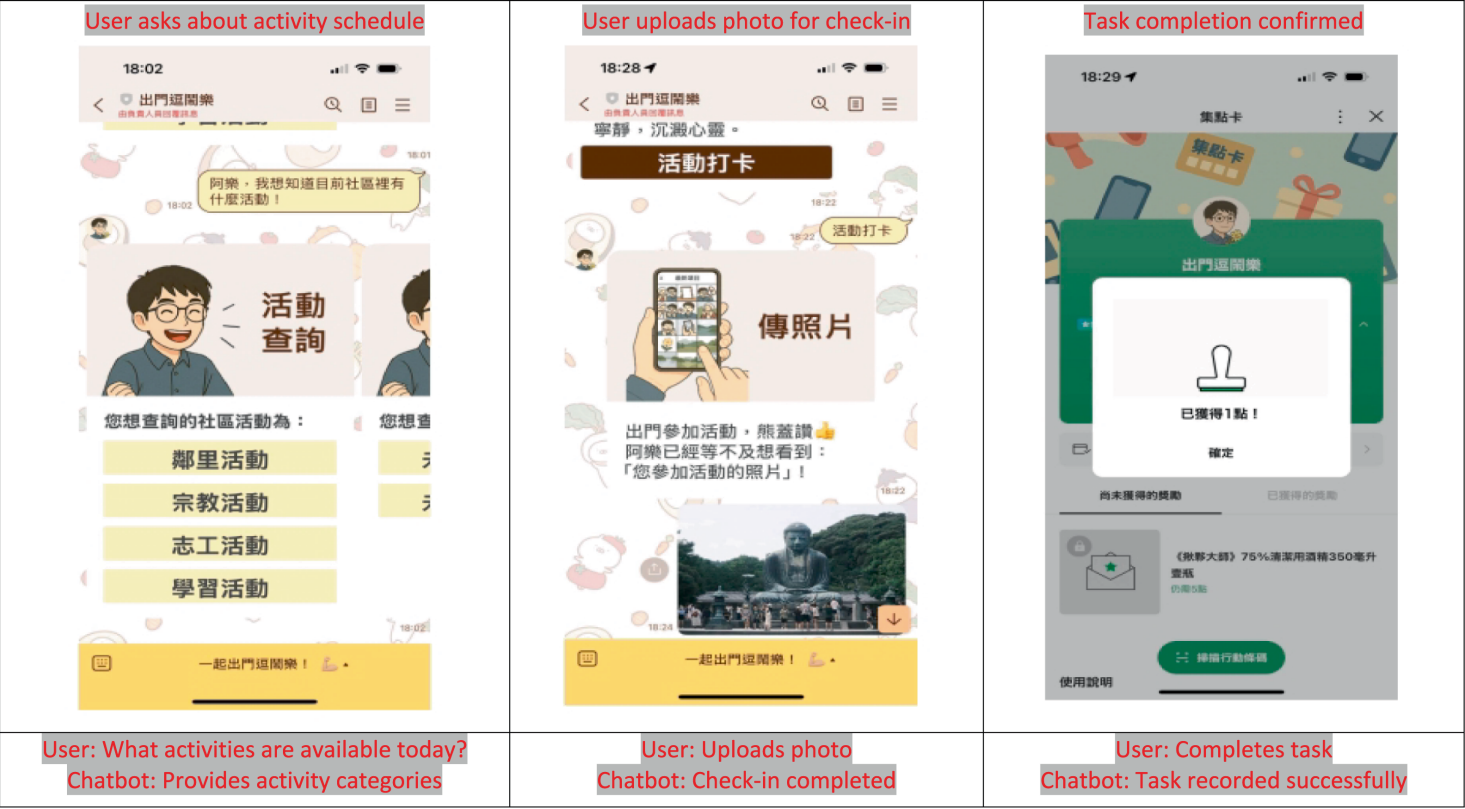
Example user–chatbot interactions in the LINE-based chatbot system used in this study.

A schematic diagram illustrating the structure and functional modules of the LINE-based chatbot system is presented in Figure 1. Example screenshots of the chatbot interface and interaction flow are presented in Figure 2 to provide readers with a clearer conceptual understanding of the user interaction environment evaluated in this study.

### 2.3 Functional experience with chatbot modules

Functional experience with the chatbot was assessed across the three modules: information inquiry, photo-based check-in interaction, and task achievement. Measurement items were adapted from established frameworks on digital engagement and user experience in eHealth and conversational agent research, emphasizing perceived usefulness, ease of use, clarity, and task support (13, 24, 25).

Each functional module was measured using multiple items rated on a 5-point Likert scale ranging from 1 (strongly disagree) to 5 (strongly agree). Composite mean scores were calculated for each module, with higher scores indicating more positive functional experience.

Internal consistency reliability was assessed using Cronbach’s alpha coefficients. All scales demonstrated excellent internal consistency (*α* = 0.932–0.948), exceeding the commonly accepted threshold of 0.70.

Because the constructs were operationalized as composite experience dimensions rather than latent theoretical constructs, the analysis focused on scale reliability and regression-based associations rather than confirmatory factor modeling.

### 2.4 Overall system use experience

Overall system use experience was measured as a global evaluation of participants’ experiences using the LINE-based chatbot. Items assessed overall satisfaction, perceived usefulness, and willingness to continue using the system. Responses were recorded using the same 5-point Likert scale, and a composite mean score was computed, with higher scores indicating more favorable overall system use experience.

### 2.5 Data collection procedure

Data were collected using a paper-based questionnaire administered on-site with assistance from trained staff when necessary. Participants were informed of the study purpose, assured of anonymity, and advised that participation was voluntary. To minimize response bias, participants were encouraged to answer all questions based on their actual experiences using the chatbot rather than expectations or hypothetical scenarios.

### 2.6 Ethical considerations

This study was approved by the Institutional Review Board of Tainan Municipal Hospital (IRB No. 1140301). All procedures were conducted in accordance with relevant ethical guidelines and regulations. Written informed consent was waived due to the anonymous nature of the questionnaire survey and the minimal risk posed to participants.

In line with best practices for conversational agent research, particular attention was given to data privacy and confidentiality. No personally identifiable information was collected, and all survey data were stored securely with restricted access. Although the chatbot operated on a commercial messaging platform, the present study focused exclusively on self-reported user experience data rather than chatbot log data, thereby minimizing privacy-related risks (26). The reporting of this study was informed by recent recommendations emphasizing transparency in the description and evaluation of chatbot-based health interventions (14).

### 2.7 Statistical analysis

All statistical analyses were conducted using IBM SPSS Statistics (version 20). Descriptive statistics were used to summarize participants’ demographic characteristics and levels of functional experience with each chatbot module. Pearson correlation analyses were performed to examine associations among functional experience variables and overall system use experience.

Multiple linear regression analysis was conducted to assess the associations between chatbot functional experiences (information inquiry, photo-based check-in interaction, and task achievement) and overall system use experience. Prior to regression analysis, assumptions of normality, linearity, independence of errors, and multicollinearity were examined and met. Statistical significance was set at a two-tailed *p* value of less than 0.05. Because all variables were measured using self-reported questionnaires collected at a single time point, potential common method variance was considered when interpreting the results.

## 3 Results

### 3.1 Participant characteristics

Table 1 summarizes the demographic characteristics of the 299 older adults included in the analysis. More than half of the participants were male (57.5%), and the majority were aged between 65 and 74 years, with the largest proportion in the 65–69 age group (39.1%). Participants aged 60–64 years accounted for 20.7%, while those aged 75 years and older represented a smaller proportion of the sample.

**Table 1:** Characteristics of the study participants (*N* = 299).

Variable	*n*	%
Gender		
Male	172	57.5
Female	127	42.5
Age group (years)		
60–64	62	20.7
65–69	117	39.1
70–74	80	26.8
75–79	38	12.7
≥80	2	0.7
Education level		
Illiterate/primary school	23	7.7
Junior high school	34	11.4
Senior high school	109	36.5
College or above	133	44.5

Percentages may not total 100% due to rounding.

Regarding educational attainment, the largest proportion of participants had completed college-level education or above (44.5%), followed by senior high school or vocational education (36.5%). A smaller proportion reported junior high school (11.4%) or elementary-level education or below (7.7%). Overall, the sample reflects community-dwelling older adults with relatively diverse educational backgrounds and sufficient digital literacy to engage with a LINE-based chatbot.

### 3.2 Descriptive statistics and reliability of study variables

Table 2 presents the descriptive statistics and internal consistency reliability of the study variables. Participants reported moderate to moderately high levels of functional experience across all chatbot modules. Among the three functional modules, task achievement experience had the highest mean score (*M* = 3.40, SD = 0.54), followed by overall system use experience (*M* = 3.35, SD = 0.54) and information inquiry experience (*M* = 3.28, SD = 0.54). Photo-based check-in experience had a slightly lower mean score (*M* = 3.04, SD = 0.54), though still above the scale midpoint.

**Table 2:** Descriptive statistics and reliability of study variables.

Variable	Items	*M*	SD	Cronbach’s *α*
Information inquiry experience	10	3.28	0.54	0.932
Photo check-in experience	10	3.04	0.54	0.937
Task achievement experience	10	3.40	0.54	0.938
Overall system use experience	11	3.35	0.54	0.948

All scales demonstrated excellent internal consistency reliability, with Cronbach’s *α* values ranging from 0.932 to 0.948, indicating satisfactory measurement quality for subsequent analyses.

### 3.3 Correlations among functional experience variables

As shown in Table 3, Pearson correlation analyses revealed significant positive correlations among all functional experience variables (*p* < 0.01). Information inquiry experience was strongly correlated with task achievement experience (*r* = 0.864) and overall system use experience (*r* = 0.817). Task achievement experience also showed a strong positive correlation with overall system use experience (*r* = 0.857). Photo-based check-in experience exhibited moderate to strong correlations with the other functional modules and overall system use experience (*r* values ranging from 0.656 to 0.767).

**Table 3:** Pearson correlations among chatbot functional experience variables (*N* = 299).

Variable	1	2	3	4
1. Information inquiry experience	1			
2. Photo-based check-in experience	0.767^**^	1		
3. Task achievement experience	0.864^**^	0.666^**^	1	
4. Overall system use experience	0.817^**^	0.656^**^	0.857^**^	1

*p* < 0.01 (two-tailed).

All correlations were statistically significant, and 95% confidence intervals did not include zero.

Although the correlations were substantial, none exceeded commonly accepted thresholds for severe multicollinearity, thereby supporting the inclusion of all functional modules in the regression analysis. Because these functional modules are integrated components of the same chatbot system, moderate correlations among user experience measures were expected.

### 3.4 Multiple linear regression analysis

Table 4 presents the results of the multiple linear regression analysis predicting overall system use experience. The regression model was statistically significant and explained a large proportion of variance in overall system use experience (*R*^2^ = 0.760, adjusted *R*^2^ = 0.757, *F* (3, 295) = 310.72, *p* < 0.001).

**Table 4:** Multiple linear regression analysis predicting overall system use experience (*N* = 299).

Predictor	*B*	SE	*β*	*t*	*p*	VIF
Constant	0.302	0.103	–	2.940	0.004	–
Information inquiry experience	0.253	0.066	0.252	3.827	<0.001	5.34
Photo-based check-in experience	0.067	0.045	0.066	1.483	0.139	2.43
Task achievement experience	0.592	0.056	0.595	10.490	<0.001	3.95

Multicollinearity diagnostics indicated acceptable levels, with variance inflation factors ranging from 2.43 to 5.34 and tolerance values within acceptable ranges.

Dependent variable: Overall system use experience.

Model statistics: *R*^2^ = 0.760; Adjusted *R*^2^ = 0.757; *F*(3, 295) = 310.72; *p* < 0.001.

VIF, variance inflation factor.

Multicollinearity diagnostics indicated acceptable levels, with variance inflation factors (VIFs) below the commonly suggested threshold of 10 and tolerance values within acceptable ranges, suggesting no serious multicollinearity among predictors.

Among the three chatbot functional modules, task achievement experience was the strongest predictor of overall system use experience (*β* = 0.595, *p* < 0.001). Information inquiry experience was also positively and significantly associated with overall system use experience (*β* = 0.252, *p* < 0.001). In contrast, photo-based check-in experience was not significantly associated with overall system use experience after controlling for the other functional modules (*β* = 0.066, *p* = 0.139).

These findings indicate that task-oriented and information-focused chatbot functions contribute more strongly to older adults’ overall system use experience than photo-based interactive features.

## 4 Discussion

### 4.1 Main findings

This study examined older adults’ functional experiences with a LINE-based chatbot designed to facilitate engagement with community activity programs and explored how different chatbot functions were associated with overall system use experience. The findings provide several important insights into the role of function-specific chatbot design in shaping older adults’ engagement.

First, task achievement experience emerged as the strongest predictor of overall system use experience. This result suggests that task-oriented and goal-focused chatbot functions play a central role in determining how older adults evaluate their overall interaction with the system. This finding is consistent with prior evidence indicating that structured, purpose-driven conversational agents are more likely to sustain engagement and perceived usefulness, particularly in health and behavior-related interventions (15, 16). Systematic reviews have further emphasized that chatbots facilitating concrete actions, progress tracking, and goal completion tend to support stronger user engagement and perceived usefulness than purely conversational or informational systems (4, 6).

From a gerontological perspective, task achievement functions may be especially salient for older adults because they provide clear objectives, immediate feedback, and a sense of accomplishment. Prior qualitative and participatory design studies have shown that older users value clarity, structure, and perceived purpose in digital interventions, and that ambiguous or overly exploratory interactions may reduce motivation or increase cognitive burden (20, 21). In the context of community engagement, task-oriented chatbot features may therefore reinforce routine participation and support engagement with community-related activities by transforming abstract engagement goals into manageable and meaningful actions, as reflected in more favorable system use experiences rather than direct behavioral outcomes.

Second, information inquiry experience was also significantly and positively associated with overall system use experience, although its effect size was smaller than that of task achievement. This finding aligns with previous research demonstrating that conversational agents function effectively as information facilitators, particularly when they reduce access barriers and provide timely, relevant responses (7, 13). For older adults, reliable access to community-related information through familiar messaging platforms may enhance perceived usefulness and trust in the system, thereby contributing to a more favorable overall experience. However, information provision alone appears insufficient to fully explain engagement, underscoring the importance of coupling informational functions with actionable components.

In contrast, photo-based check-in experience was not significantly associated with overall system use experience after controlling for other functional modules. Although this interactive feature demonstrated moderate correlations with other functional experiences, its unique contribution to overall system use was limited. This finding echoes prior research suggesting that socially oriented or media-based chatbot interactions do not consistently translate into stronger overall engagement, particularly among older users (17, 18). One possible explanation is that photo-based interactions may impose additional cognitive or technical demands, such as operating smartphone cameras or managing uploads, which can diminish perceived ease of use. Earlier studies have reported that older adults are more sensitive to interaction complexity and may disengage from features perceived as non-essential or effortful (1, 11).

Importantly, the present findings do not suggest that photo-based or interactive features lack value altogether. Rather, they indicate that such functions may serve a supplementary role rather than acting as primary drivers of overall system use experience. This interpretation is consistent with prior reviews emphasizing that interactive or social chatbot features may enhance enjoyment or emotional connection but are most effective when integrated with clear functional goals and practical utility (2, 3).

Taken together, these results reinforce recent calls for moving beyond treating chatbots as homogeneous interventions and instead adopting a function-oriented evaluation framework (14). By empirically demonstrating that different chatbot functions contribute unequally to overall system use experience among older adults, this study highlights the importance of prioritizing task-oriented and information-focused design elements when developing chatbot-based interventions. At the same time, the relatively high explained variance observed in the regression model may partially reflect shared perceptual variance arising from self-reported measures collected at a single time point.

### 4.2 Comparison with prior research on task-oriented chatbot functions

The strong association between task achievement experience and overall system use experience observed in this study is consistent with prior research emphasizing the effectiveness of goal-oriented and action-driven conversational agents. Randomized controlled trials and systematic reviews have shown that chatbots designed to support concrete tasks—such as goal tracking, reminders, and stepwise guidance—are more likely to sustain engagement and produce meaningful behavioral outcomes (4, 6, 15). Similarly, meta-analytic evidence suggests that task-focused conversational agents outperform purely conversational systems in promoting adherence and continued use across health-related contexts (16).

However, the present study adds nuance to this literature by demonstrating that task achievement functions remain central even in non-clinical, community-based settings focused on social participation. While prior studies have primarily examined task-oriented chatbots in domains such as smoking cessation, chronic disease management, or lifestyle modification, the current findings suggest that structured task support may also be important for enhancing engagement with chatbot-based systems used in community activity contexts among older adults.

### 4.3 Information inquiry functions in relation to existing evidence

The significant, yet comparatively smaller, effect of information inquiry experience aligns with prior findings indicating that conversational agents are effective information facilitators but may have limited impact on sustained engagement when used in isolation. Reviews of digital health chatbots have consistently reported that information provision enhances perceived usefulness and trust but does not necessarily translate into long-term system use unless paired with interactive or goal-oriented components (7, 13).

In studies involving older adults, access to reliable and easily retrievable information through familiar platforms has been shown to reduce informational barriers and improve initial acceptance of AI-based systems (10, 11). The present findings support this literature by confirming the importance of information inquiry functions, while simultaneously suggesting that their impact on overall system use is secondary to task-oriented features.

### 4.4 Reconsidering the role of interactive and media-based features

The lack of a significant association between photo-based check-in experience and overall system use experience contrasts with some qualitative studies that emphasize the social and emotional value of interactive chatbot features. For example, prior qualitative and mixed-method studies have suggested that media-based interactions and social exchanges may enhance enjoyment or perceived connectedness among older users (2, 18).

Nevertheless, the present findings are consistent with other empirical evidence indicating that increased interactivity does not necessarily lead to improved usability or sustained engagement, particularly among older adults who may be more sensitive to cognitive and technical demands (1, 17). Research on voice assistants and smart speakers similarly suggests that while social interaction features can be appealing, users prioritize simplicity, reliability, and functional utility over novelty (11, 12).

Rather than contradicting earlier qualitative findings, the current results help clarify their boundaries by showing that interactive features may function best as complementary elements rather than primary drivers of system use. This distinction highlights the value of quantitatively testing assumptions derived from qualitative insights.

### 4.5 Contribution to methodological and reporting frameworks

By empirically demonstrating differential effects of chatbot functions, this study responds directly to recent calls for more granular evaluation and transparent reporting of conversational agent design. The CHART reporting guideline emphasizes the need to clearly describe chatbot functionalities and to examine how specific design elements relate to outcomes. Similarly, recent systematic reviews of large language model–based chatbots have highlighted the lack of function-level analyses as a key limitation of existing research (14).

The function-oriented approach adopted in this study therefore contributes methodologically by offering an empirical framework that complements existing qualitative and review-based evidence. This approach may help bridge the gap between descriptive accounts of chatbot design and quantitative evaluations of user experience.

### 4.6 Practical implications

From a practical perspective, the findings of this study provide important implications for the design and implementation of chatbot-based interventions targeting older adults. First, the strong association between task achievement functions and overall system use experience suggests that chatbot systems should prioritize goal-oriented and action-driven features. Designers and developers may consider incorporating functionalities such as task completion tracking, reminders, and progress feedback to enhance user engagement and perceived usefulness.

Second, while information inquiry functions contribute positively to system use experience, their impact appears to be more limited when implemented alone. This indicates that informational features should be integrated with actionable components to support both accessibility and user engagement. Ensuring that information is presented clearly and delivered through familiar platforms, such as LINE, may further enhance usability among older adult populations.

Third, the findings suggest that interactive features, such as photo-based check-in, may play a supplementary rather than central role in shaping system use experience. Developers should therefore carefully evaluate the complexity and necessity of such features, particularly for older users who may be more sensitive to cognitive and technical demands. Simplifying interaction processes and minimizing unnecessary steps may help reduce user burden and improve overall system acceptance.

From a program implementation perspective, embedding chatbot systems within widely used communication platforms may facilitate adoption and sustained engagement in community-based settings. These findings highlight the importance of aligning chatbot functionality with users’ practical needs, preferences, and digital literacy levels when designing interventions for older adults.

### 4.7 Strengths and limitations

This study has several notable strengths that contribute to the existing literature on chatbot-based interventions for older adults. First, rather than treating chatbot use as a single, undifferentiated construct, this study adopted a function-oriented analytical framework. By disaggregating chatbot use into information inquiry, photo-based check-in interaction, and task achievement modules, the study provides more granular insights into how specific chatbot functions shape overall system use experience. This approach responds to prior calls in the literature for more detailed evaluations of chatbot functionality and design components.

Second, the study examined chatbot use within a real-world, community-based context using a widely adopted messaging platform. The use of LINE, a familiar and commonly used application among older adults in Taiwan, enhances the ecological validity of the findings. Unlike experimental settings that rely on newly introduced or unfamiliar systems, this study reflects older adults’ experiences with a chatbot embedded in their everyday communication environment, thereby increasing the practical relevance of the results for community engagement and active aging initiatives.

Third, the study demonstrated strong measurement quality, with all functional experience scales exhibiting excellent internal consistency reliability. The analytical strategy, which combined descriptive statistics, correlation analysis, and multiple linear regression, allowed for a comprehensive examination of relationships among chatbot functions while accounting for their intercorrelations. The large proportion of explained variance in overall system use experience further suggests that the selected functional modules capture key dimensions of older adults’ chatbot experiences.

Despite these strengths, several limitations should be acknowledged. First, the cross-sectional design precludes causal inference. Although significant associations were identified between functional experiences and overall system use experience, it is not possible to determine the directionality of these relationships. Longitudinal or experimental studies are needed to clarify causal pathways and to examine how functional experiences evolve over time.

Second, the potential influence of common method variance should be considered because all variables were measured using self-reported questionnaires collected at a single time point. Although multicollinearity diagnostics suggested acceptable levels of predictor overlap, future studies could incorporate objective system usage data or multi-source measurements to further reduce potential method bias.

Third, the study relied on self-reported data, which may be subject to recall bias or social desirability bias. Participants’ responses reflect their perceived experiences rather than objective usage metrics. While self-reported user experience is a commonly accepted outcome in digital health and human–computer interaction research, future studies could integrate system log data or behavioral indicators to complement subjective assessments.

Finally, the generalizability of the findings may be limited by the study context and sample characteristics. Participants were community-dwelling older adults in Taiwan who had prior experience using the LINE messaging platform. This may introduce a degree of selection bias toward individuals who are already familiar with digital communication tools. Older adults with lower levels of digital literacy or those in different cultural or technological contexts may exhibit different patterns of chatbot use and functional preferences. Caution is therefore warranted when extrapolating the findings to other populations or platforms.

## 5 Conclusion

This study examined older adults’ functional experiences with a LINE-based chatbot designed to facilitate engagement with community activity programs and explored how different chatbot functions are associated with overall system use experience. By adopting a function-oriented analytical framework, the study provides empirical evidence suggesting that not all chatbot features contribute equally to older adults’ engagement with digital systems.

The findings indicate that task achievement functions are more strongly associated with overall system use experience, followed by information inquiry functions, whereas photo-based interactive features do not independently predict system use experience after accounting for other functional components. These results underscore the importance of prioritizing goal-oriented and action-focused chatbot design when developing digital interventions for older adults.

Beyond contributing to the growing literature on conversational agents in digital health, this study extends existing evidence by situating chatbot use within the context of digital engagement with community activity programs. The use of a widely adopted messaging platform enhances the practical relevance of the findings and highlights the potential of platform-embedded chatbots as scalable digital tools for supporting engagement with community activity programs among older adults.

Overall, the study suggests that future chatbot-based interventions for older adults may benefit from moving beyond generic or entertainment-driven designs and instead focusing on functional elements that align with users’ needs for clarity, purpose, and meaningful engagement. By informing function-specific design and evaluation strategies, this research may contribute to the development of more effective, user-centered chatbot systems for older adult populations.

## Data Availability

The original contributions presented in the study are included in the article/supplementary material, further inquiries can be directed to the corresponding author.

## Appendix A. Measurement items used in the questionnaire

Participants rated all items using a five-point Likert scale ranging from 1 (strongly disagree) to 5 (strongly agree).

### A1. Information inquiry experience (10 items)

1. I intend to use the community activity inquiry function.
2. I am willing to continue using the community activity inquiry function.
3. Within the next month, I plan to use the community activity inquiry function.
4. Using the community activity inquiry function makes it easier for me to participate in community activities.
5. Using the community activity inquiry function improves the efficiency of my participation in community activities.
6. The community activity inquiry function helps me manage my participation in community activities.
7. The interface of the community activity inquiry function is clear and easy to understand.
8. Using the community activity inquiry function does not require much mental effort.
9. The community activity inquiry function is easy to learn and operate.
10. I can easily participate in community activities using the community activity inquiry function.

### A2. Photo check-in mechanism experience (10 items)

1. I intend to use the photo check-in mechanism.
2. I am willing to continue using the photo check-in mechanism.
3. Within the next month, I plan to use the photo check-in mechanism.
4. Using the photo check-in mechanism makes it easier for me to participate in community activities.
5. Using the photo check-in mechanism improves the efficiency of my participation in community activities.
6. The photo check-in mechanism supports my social participation behavior.
7. The interface of the photo check-in mechanism is clear and easy to understand.
8. Using the photo check-in mechanism does not require much mental effort.
9. The photo check-in mechanism is easy to learn and operate.
10. I can easily participate in community activities using the photo check-in mechanism.

### A3. Task achievement mechanism experience (10 items)

1. I intend to use the task achievement mechanism.
2. I am willing to continue using the task achievement mechanism.
3. Within the next month, I plan to use the task achievement mechanism.
4. Using the task achievement mechanism makes it easier for me to participate in community activities.
5. Using the task achievement mechanism improves the efficiency of my participation in community activities.
6. The task achievement mechanism helps me participate in community activities.
7. The interface of the task achievement mechanism is clear and easy to understand.
8. Using the task achievement mechanism does not require much mental effort.
9. The task achievement mechanism is easy to learn and operate.
10. I can easily participate in community activities using the task achievement mechanism.

### A4. Overall system use experience (11 items)

1. I intend to use “Go Out and Have Fun” (the chatbot system).
2. I am willing to continue using “Go Out and Have Fun.”
3. Within the next month, I plan to use “Go Out and Have Fun.”
4. Using “Go Out and Have Fun” makes it easier for me to participate in community activities.
5. Using “Go Out and Have Fun” allows me to obtain more comprehensive services related to community activities.
6. Using “Go Out and Have Fun” improves the efficiency of my participation in community activities.
7. “Go Out and Have Fun” helps me participate in community activities.
8. The interface of “Go Out and Have Fun” is clear and easy to understand.
9. Using “Go Out and Have Fun” does not require much mental effort.
10. “Go Out and Have Fun” is easy to learn and operate.
11. I can easily participate in community activities using the chatbot system.
